# Bilateral Renal Cell Carcinoma in the Horseshoe Kidney With Metastasis in Gallbladder, Pancreas and Duodenum: A Report of a Rare Case and Literature Review

**DOI:** 10.7759/cureus.58363

**Published:** 2024-04-16

**Authors:** Chetana Ratnaparkhi, Avinash Dhok, Neeraj Kumar, Shantanu Pande, Sakshi Jeswani

**Affiliations:** 1 Radiodiagnosis, All India Institute of Medical Sciences, Nagpur, Nagpur, IND; 2 Radiodiagnosis and Interventional Radiology, All India Institute of Medical Sciences, Nagpur, Nagpur, IND; 3 Radiodiagnosis, Shree Mangala Hospital, Bilaspur, IND; 4 Nuclear Medicine, All India Institute of Medical Sciences, Nagpur, Nagpur, IND

**Keywords:** duodenal metastasis, pancreas metastasis, gall bladder metastasis, horseshoe kidney, renal cell carcinoma

## Abstract

Horseshoe kidney is the most common renal fusion anomaly and is associated with various complications, ranging from infections to neoplasms. While renal cell carcinoma (RCC) is the most frequent renal neoplasm in adults, its occurrence in a horseshoe kidney is rare, and bilateral involvement is rarer. Furthermore, RCC metastasizing to organs is known and rare sites of metastasis are also documented. The report presents a unique case of bilateral RCC in a horseshoe kidney with synchronous metastasis to the gallbladder, pancreas, and duodenum. This presentation, involving metastasis to these specific organs, is exceedingly uncommon, making it a rarest of rare possibilities. The current case report underscores the importance of vigilant monitoring and comprehensive evaluation in patients with horseshoe kidneys, as they may be predisposed to unusual complications like RCC and rare site metastasis.

## Introduction

Horseshoe kidney is the commonest renal fusion anomaly and is very prone to complications like renal calculi, obstructive uropathy and renal infections. Renal cell carcinoma (RCC) is the most common renal neoplasm in adults accounting for around 90% of renal neoplasms [1]. Occurrence of RCC in horseshoe kidneys is itself a rare entity and only a few cases are mentioned in the literature [2]. Nearly one-third of RCC shows metastasis at presentation. RCC metastasizing to the liver, lung, brain and bones is known. However, metastasis in the gallbladder and pancreas is very rare, and only a few cases have been mentioned previously. The current report presented a case of RCC in the horseshoe kidney with simultaneous metastasis in the gallbladder, pancreas and peritoneum.

## Case presentation

A 57-year-old female presented to the urology department with a chief complaint of left lumbar region pain for five days. The patient had an episode of right lumbar region pain and hematuria six years back. She underwent ultrasound and contrast-enhanced computed tomography (CT) of the abdomen which revealed a horseshoe kidney with a large, enhancing heterogeneously soft tissue density mass originating from the interpolar region of the right renal moiety, suggestive of RCC. Another 1.1x1cm sized solid, soft tissue density, moderately enhancing lesion was also seen in the interpolar region of the left renal moiety. After successful pre-operative embolization of the right renal moiety mass, a right radical nephrectomy was performed. Post-surgical histopathological examination confirmed the diagnosis of clear cell variety of RCC.

Radical nephrectomy was followed by radical radiotherapy which also included the left renal moiety lesion. On six monthly interval follow-ups, the left renal moiety lesion did not show any significant change in size. Due to some personal issues, the patient could not do regular follow up. During her current visit owing to similar complaints, serum creatinine, sodium, potassium, hemogram, liver function tests and abdominal ultrasound were done. All blood parameters were in the normal range.

Abdominal ultrasound was performed on GE-LOGIQ-P9 (GE Healthcare, Chicago, USA) with the convex probe of frequency 4MHz and linear probe with a frequency of 9MHz. Ultrasound showed a predominantly exophytic left renal mass with moderate vascularity. No calcification was noted within the mass. Also, there were heterogenous echogenicity peripancreatic and mesenteric lesions which showed moderate vascularity. There was no thrombosis in the inferior vena cava (IVC) or left renal vein. Ultrasound findings suggested RCC and hence, the patient was further subjected to CT urography.

CT scan was done on a Siemens 256 slice dual-energy dual-source CT scanner (Siemens Healthineers, Erlangen, Germany) in preset CT urography format. Intravenous iohexol, 350 mg/mL in the dose of 1.5 mg/kg, was used as contrast. Screening of thorax for metastasis was also done in the same setting.

**Figure 1:**
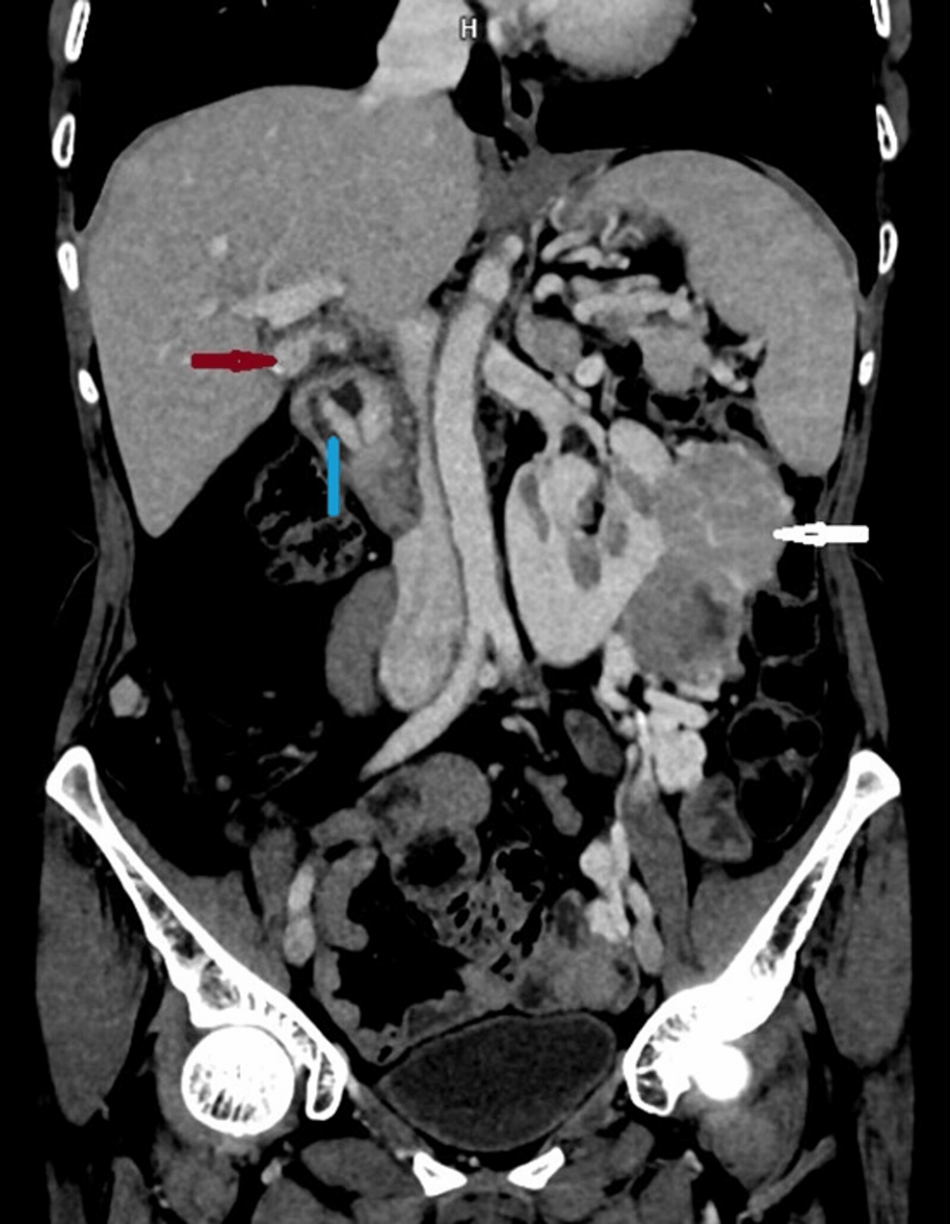
Coronal post-contrast computed tomography (CT) at the level of the left kidney Coronal contrast-enhanced CT shows an exophytic mass in the left moiety of the horseshoe kidney (white arrow). Also noted is an enhancing lesion in the gallbladder (red arrow) and duodenum (blue arrow).

CT urography showed an exophytic, smoothly marginated, moderately enhancing mass of size 6x6.3x7.7cm in maximum anteroposterior, transverse and cranio-caudal dimensions arising from the interpolar region of the left renal moiety. Arterial blood supply comes from branches of the left renal artery (Figure 1).

The mass was affecting the calyces. No invasion of IVC or renal vein was noted. The right-sided moiety of the horseshoe kidney was not appreciated, consistent with post-operative status.

A heterogenously enhancing mass was seen in the gallbladder neck, extending into the cystic duct (Figure 1). No intrahepatic biliary tract dilatation was present. The pancreas was enlarged with heterogeneous enhancement and some non-enhancing areas in the distal pancreatic body. Peripancreatic lymph nodes were not appreciated. The second part of the duodenum showed heterogeneously enhancing asymmetric wall thickening and loss of fat plane adjacent to the head of the pancreas (Figure 1). Lesions in the gallbladder neck, pancreatic body and duodenum showed similar imaging characteristics. No lesion was observed in the lung or visualised bones. On CT urography, RCC stage IV in left renal moiety with metastasis in gallbladder, pancreas and duodenum was suggested.

**Figure 2:**
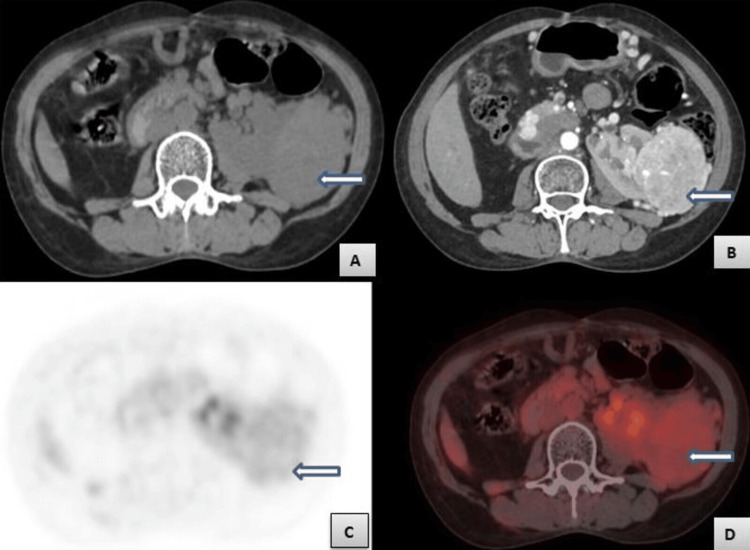
Axial computed tomography (CT) and positron emission tomography-computed tomography (PET-CT) at the level of kidney Figure 2A - Axial pre-contrast CT image showing tissue density mass in the left renal moiety (white arrow). Figure 2B - Axial post-contrast CT image in arterial phase showing enhancing mass in the left renal moiety (white arrow). Figures 2C-2D - Axial PET and PET-CT images showing diffuse uptake of FDG by the lesion in the left renal moiety (white arrows) with a standardized uptake value of 4.1. FDG: fluorodeoxyglucose

**Figure 3:**
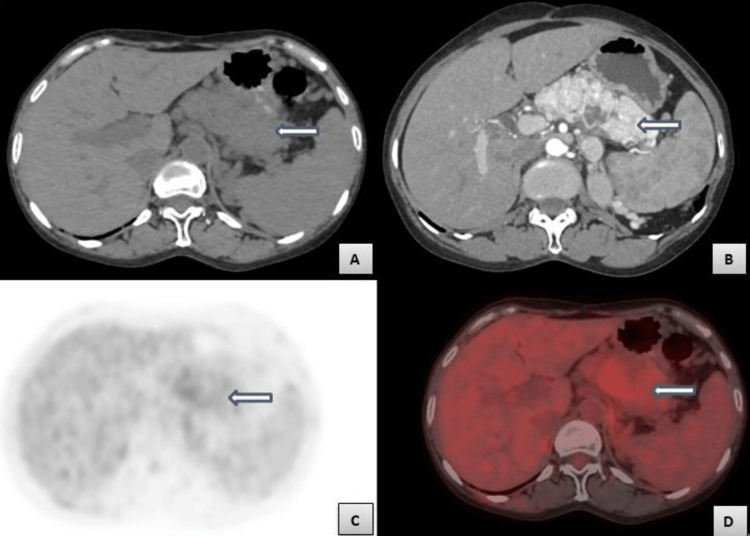
Axial computed tomography (CT) and positron emission tomography-computed tomography (PET-CT) at the level of pancreas Figure 3A - Axial pre-contrast CT image showing soft tissue density mass in the distal body of the pancreas (white arrow). Figure 3B - Axial post-contrast, portal phase, CT image showing heterogeneously enhancing mass in the distal pancreatic body (white arrow). Figures 3C-3D - Axial PET and PET-CT images showing diffuse uptake by the pancreatic lesion (white arrows) with a standardized uptake value of 4.13.

**Figure 4:**
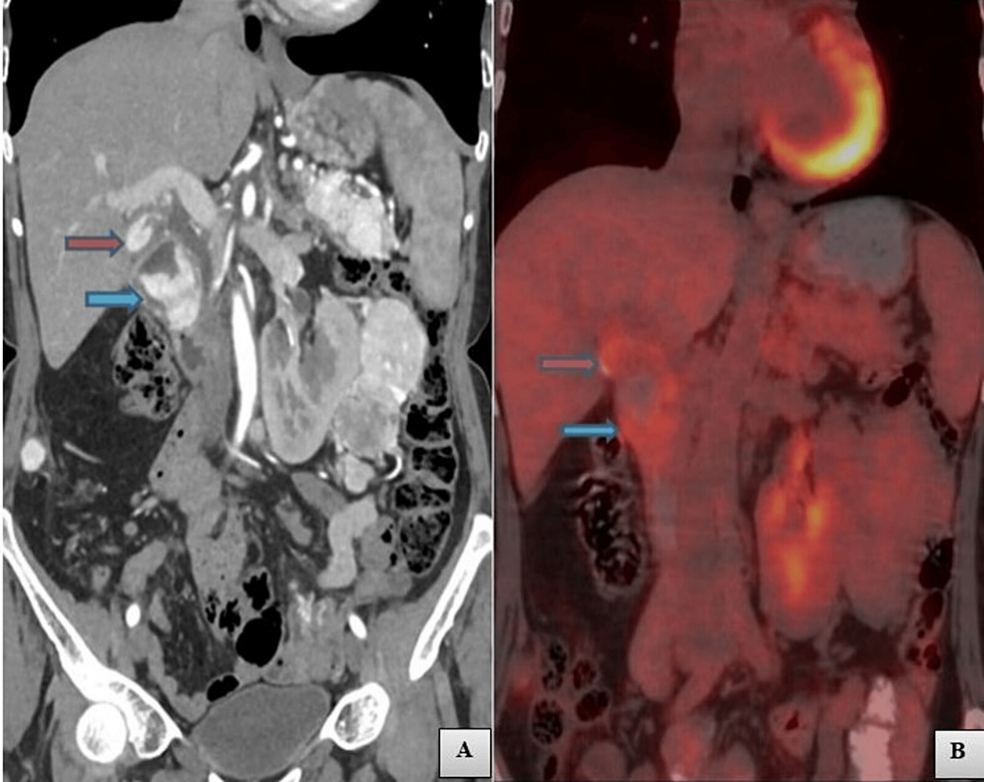
Coronal computed tomography (CT) and positron emission tomography-computed tomography (PET-CT) Figure 4A - Coronal post-contrast CT image showing enhancing lesions in the gallbladder (red arrow) and duodenum (blue arrow). Figure 4B - Coronal PET-CT image showing a significant uptake by the lesion in the gallbladder (red arrow) and duodenum (blue arrow) showing standardized uptake values of 4.5 and 5.1 respectively.

To assess the metabolic nature of the mass, fluoro-deoxy-glucose, positron-emission-tomography with CT (FDG PET-CT) of the whole body was performed. PET-CT showed avid uptake of FDG by mass in the left kidney, gallbladder, pancreas and duodenum (Figures 2-4).

Standardized uptake values (SUV) of mass in left renal moiety, gallbladder, pancreas and duodenum were 4.1, 4.5, 4.13 and 5.1 respectively. All these findings were suggestive of RCC in left renal moiety with metastasis in the gallbladder, pancreas and duodenum.

Patient treatment and follow-up

Biopsy from the masses could not be obtained due to the patient’s unwillingness. The patient was managed with chemotherapy and radiotherapy.

## Discussion

The horseshoe kidney is the most common developmental fusion anomaly accounting for approximately 1/400 live births [3]. It is very prone to complications like renal calculi formation, obstructive uropathy and infection. Though RCC accounts for nearly 3% of all malignancies in adults and 90% of renal neoplasms [1], the occurrence of RCC in horseshoe kidneys is very rare and only less than 200 cases are mentioned in the literature [2]. However, the risk of RCC in horseshoe kidneys is the same as compared to the normal population [4].

Imaging is vital in the diagnosis of RCC. On ultrasound, RCC is a solid mass with or without a cystic component. CT scan is helpful in the diagnosis and staging of RCC. On pre-contrast CT, RCC is seen as soft tissue density mass. Calcification is seen in approximately 30% of RCC [5]. RCC shows significant post-contrast enhancement.

MRI helps in the diagnosis and characterization of histological types depending on the T2 characteristics of the lesion. The clear cell type is hyperintense and the papillary type is hypointense [6]. On post-gadolinium scan, RCC shows prompt enhancement in the arterial phase. FGD-PET-CT has limited value in diagnosing primary RCC. However, it is crucial in the diagnosis of recurrence and treatment response in follow-up.

RCC metastasis is known and nearly one-third of newly diagnosed cases already have synchronous metastatic disease at presentation [7]. In cases of metastasis, the survival rate of five years reduces to 10-15% [8]. Common organs of RCC metastasis are liver, lung, bone, adrenal gland, brain and contralateral kidney.

RCC metastasis in the pancreas is rare and reported only in 1.6-11% of autopsies [9]. However, in pancreatic metastasis cases, RCC is the most common primary tumour, followed by lung cancer, breast cancer, colon cancer and melanoma [10]. On contrast-enhanced CT scans, these lesions also show early enhancement as compared to the rest of the pancreatic parenchyma and merge with the pancreatic parenchyma in delayed phases, similar to RCC [9,10]. In the current case, the pancreas was enlarged with heterogeneous enhancement and few hetero-enhancing lesions in the distal pancreatic body, showing an enhancement pattern similar to RCC.

Gallbladder metastasis is reported in less than 1% RCC [7]. In the literature, limited cases of RCC metastasizing to the gallbladder are reported. In a literature review by Costa Neves et al., they studied 52 patients of gallbladder metastasis in RCC, most of them were elderly males with incidentally detected lesions while scanning for primary malignancy or follow-up [7]. In the current case, the patient was a middle-aged female without any symptoms pertaining to gallbladder pathology. On imaging, these lesions showed features similar to primary lesions, i.e. significant enhancement in the arterial phase.

According to the literature, in horseshoe kidney, the commonest cell type is the transitional cell type, as the predisposing factor is chronic urinary tract infection [4]. However, in the present case, it was clear cell variety on histopathology of the right renal moiety mass. The role of FDG-PET-CT is important in the follow-up of cases. In the current case, the renal lesion showed uptake of FDG. Gallbladder, pancreatic and duodenal lesions showed similar uptake and SUV values, which suggested metastasis of RCC to these organs.

The present case is exceptional in that, it had bilateral RCC in the horseshoe kidney who underwent partial nephrectomy six years back with synchronous metastasis in the gallbladder, pancreas and duodenum. Metastasis to the gallbladder, pancreas and duodenum from any malignancy is uncommon. Moreover, simultaneous metastasis to these organs is the rarest of rare possibilities. Although RCC is an aggressive tumor with approximately one-third of patients having either distant metastasis or significant loco regional spread at presentation, no definite pattern of metastasis has been defined yet [7].

## Conclusions

Bilateral RCC in horseshoe kidney with synchronous metastasis in the gallbladder, pancreas and duodenum is a rare possibility. To accurately identify the presence and extent of metastatic disease in RCC, thorough evaluation with imaging techniques like ultrasound, contrast-enhanced CT, MRI and PET-CT is needed. Detecting metastatic lesions at unusual locations is crucial for treatment planning and predicting outcomes in RCC. With advancements in medical knowledge and technologies, medical professionals should be vigilant and think of atypical presentations of metastasis. Collaborative efforts among different specialities along with advanced imaging technologies can help in early detection and treatment of rare site metastasis.
